# Mutational Pattern in Multiple Pulmonary Nodules Are Associated With Early Stage Lung Adenocarcinoma

**DOI:** 10.3389/fonc.2020.571521

**Published:** 2021-02-19

**Authors:** Shao-wei Dong, Rong Li, Zhiqiang Cheng, Dong-cheng Liu, Jinquan Xia, Jing Xu, Shixuan Li, Jian Wang, Yongjian Yue, Yingrui Fan, Yundi Cao, Lingyun Dai, Jigang Wang, Pan Zhao, Xin Wang, Zhangang Xiao, Chen Qiu, Guang-suo Wang, Chang Zou

**Affiliations:** ^1^ Clinical Medical Research Centre, The First Affiliated Hospital, Southern University of Science and Technology, Shenzhen People’s Hospital, Shenzhen, China; ^2^ Shenzhen Public Service Platform on Tumor Precision Medicine and Molecular Diagnosis, Shenzhen, China; ^3^ Department of Oncology, Taikang Xianlin Drum Tower Hospital, Nanjing University School of Medicine, Nanjing, China; ^4^ Department of Pathology, The First Affiliated Hospital, Southern University of Science and Technology, Shenzhen People’s Hospital, Shenzhen, China; ^5^ Department of Thoracic Surgery, The First Affiliated Hospital, Southern University of Science and Technology, Shenzhen People’s Hospital, Shenzhen, China; ^6^ Department of Respiratory and Critical Medicine, Shenzhen People’s Hospital, Second Clinical Medical College of Jinan University, Shenzhen, China; ^7^ Department of Geriatrics, The First Affiliated Hospital, Southern University of Science and Technology, Shenzhen People’s Hospital, Shenzhen, China; ^8^ Department of Biomedical Sciences, City University of Hong Kong, Hong Kong, Hong Kong; ^9^ Key Laboratory of Medical Electrophysiology of Ministry of Education, School of Pharmacy, Southwestern Medical University, Luzhou, China

**Keywords:** pulmonary nodules, whole-exome sequencing, mutation, functional analysis, pathway analysis

## Abstract

The clinical significance of mutation in multiple pulmonary nodules is largely limited by single gene mutation-directed analysis and lack of validation of gene expression profiles. New analytic strategy is urgently needed for comprehensive understanding of genomic data in multiple pulmonary nodules. In this study, we performed whole exome sequencing in 16 multiple lung nodules and 5 adjacent normal tissues from 4 patients with multiple pulmonary nodules and decoded the mutation information from a perspective of cellular functions and signaling pathways. Mutated genes as well as mutation patterns shared in more than two lesions were identified and characterized. We found that the number of mutations or mutated genes and the extent of protein structural changes caused by different mutations is positively correlated with the degree of malignancy. Moreover, the mutated genes in the nodules are associated with the molecular functions or signaling pathways related to cell proliferation and survival. We showed a developing pattern of quantity (the number of mutations/mutated genes) and quality (the extent of protein structural changes) in multiple pulmonary nodules. The mutation and mutated genes in multiple pulmonary nodules are associated with cell proliferation and survival related signaling pathways. This study provides a new perspective for comprehension of genomic mutational data and might shed new light on deciphering molecular evolution of early stage lung adenocarcinoma.

## Introduction

Lung adenocarcinoma (LUAD) is the most frequent subtype of lung cancer. Even with regularly performed and thorough radiological and cytological screening in high risk populations, LUAD is usually diagnosed in its middle and late stage (1). Novel therapeutic strategies have been under active research and development to conquer LUAD, but the survival rate of patients has unfortunately kept at a low level (1). The emerging of improved cancer screening methods such as liquid biopsy holds great promise for reversing this serious situation since most of solid tumors are curable in the early stage (2). Unfortunately, this strategy is still limited by the incomplete understandings of the complicated molecular mechanisms of cancer origination and malignant progression as well as intra-tumoral heterogeneity.

Multiple pulmonary nodules (MPNs) contribute to the majority of newly diagnosed LUAD (3). Pathologically, the malignant evolution of early stage LUAD comprises four contiguous steps: atypical adenomatous hyperplasia (AAH), *in situ* pulmonary adenocarcinoma (AIS, also named as bronchioloalveolar carcinoma, BAC), minimally invasive adenocarcinoma (MIA), and invasive adenocarcinoma (IA), all of which could be well exemplified in some patients with MPNs (4). It is well characterized that AAH represents the pre-cancerous lesion of LUAD and AIS is the localized adenocarcinoma without any invasion. MIA and IA are differentiated by the tumor size. MIA is defined as single or multiple invasive neoplasia less than 5 mm and restrained in stage of IA, while IA represents invasive tumor larger than 5 mm (5).

In view of different pathological grades and clinical stages of individual adenoma loci in the same genetic background, MPNs represent an ideal model for biological characterization of molecular evolution of the early stage LUAD. A number of efforts have been made in depicting the molecular diagram of MPNs by using genome-based approaches, e.g., whole genome sequencing, whole exome sequencing (WES) and methylation analysis (6–9). However, the theoretical and clinical significance of these reported mutations are largely limited by the lack of paired validation studies due to the rarity of malignant cells as well as the paucity of neoplasia in MPNs samples (10). In addition, since the origination and malignant progression of solid tumors is a multifaced and multiple-step orchestration, these findings, mostly based on single gene change-directed analysis, needs to be carefully interpreted and reevaluated especially for clinical application.

To better understand the molecular evolution of MPNs, we performed WES in 21 tissues dissected from 4 patients with MPNs and analyzed the somatic mutations using a cell function and pathway converging-directed strategy. Mutated genes e.g., KTM2C and EGFR, and patterns of these mutations were successfully identified and characterized. No ubiquitous mutated genes or mutation loci were discovered in all malignancy or all patients. Interestingly, more mutated genes were found in IA samples than in MIA samples. KEGG (Kyoto Encyclopedia of Genes and Genomes, www.genome.jp) and GO (Gene Ontology, www.geneontology.org) analysis indicates that mutated genes tend to converge into cell proliferation and survival related signaling pathways in malignancy in comparison with adjacent normal samples. This converging tendency seems to be more obvious in IA than in MIA. Moreover, the levels of protein structural changes caused by different mutations as well as that of converged signaling pathways are higher in IA than in MIA. The levels of protein structural changes and converged signaling pathways are also higher in all malignant lesions than in adjacent normal tissues, indicating these protein molecules and pathways may be responsible for the malignant evolution of LUAD. Some specific driver genes in cancer development such as EGFR, mutations of this gene are found both in cancer lesions and adjacent normal samples, despite of more severe extent of mutations in malignancy, suggesting driver gene mutations need to be further validated particularly for clinical application. Besides, in comparison with The Cancer Genome Atlas database (TCGA), most of the mutated genes in MIA and IA identified in this study are genes functionally related to the development of lung cancers. Our findings, by function-directed analysis of WES data from MPNs, that a group of functionally converged mutations rather than a single driver gene mutation might be the triggering onset of the origination and malignant progression of lung adenocarcinoma. This study will provide new insights and new strategies to decipher the early development of lung cancer.

## Materials and Methods

### Patients and Clinical Information

Tumor samples and their matched normal tissues were obtained from four patients from Shenzhen people’s hospital from 2017 to 2019. The clinical information such as age/gender/smoking history was listed in **Supplementary Table 1**. These patients did not receive preoperative chemotherapy or radiotherapy before surgery. Tumor sizes ranged from 0.2 to 3.0 cm according to pathology reports. All patients were free of extra thoracic metastasis. Three of the patients were non-smokers, and one patient had a history of cigarette smoking. The images of CT and hematoxylin-eosin staining were reviewed by experienced pathologists to determine the histopathological subtype and were shown in **Supplementary Figure S1**. The present study was approved by the Ethics Committee of Shenzhen people’s hospital. All study procedures were performed according to the Declaration of Helsinki ethical principles. Informed consents were obtained from the patients.

### Sample Preparation

Genomic DNA from formalin-fixed paraffin-embedded (FFPE) samples was extracted using QIAamp DNA FFPE Tissue Kit (Qiagen, Valencia, CA). DNA from fresh tumor tissue and blood was extracted by DNAeasy Blood and Tissue kit (Qiagen, Valencia, CA). Genomic DNA was fragmented into ~250 bp by M220 Focused-ultrasonicator (Covaris, Inc. Woburn, MA) followed by whole genome library preparation using KAPA Hyper Prep Kit (KAPA Biosystems, Wilmington, MA). Exome capture was performed using the SureSelect Human All Exon V6 panel (Agilent Technologies, Santa Clara, CA).

### Whole Exome Sequencing (WES)

WES was performed by Geneseeq Technology Inc. (Nanjing, China) using an Illumina HiSeq4000 system. The adapters in FASTQ files were cleaned using Trimmomatic-0.36 (11). The alignment was performed using Burrows-Wheeler Aligner (12), and GRCh37 (hg19) downloaded from Ensembl website was used as reference. The exome mutations in pulmonary nodules and para-cancer (PAR) samples were compared with mutations obtained from peripheral blood mononuclear cells (PBMCs), and only somatic specific mutations were retained for further analysis.

### Converging Analysis

Converging index is defined as follows: 1) take any given two samples S1 and S2 with mutated genes from some pathways, KEGG for example 2) the numbers of mutated pathways (pathways with mutated genes) were calculated as P1 and P2; 3) the number of overlapped mutated pathways between two samples (mutated pathways occurred in both samples) is counted as P; and 4) the converging index is calculated as (P/P1)*(P/P2). The converging index in GO biological pathways and GO molecular functions is calculated using the same method.

The ranking of converged pathways/functions was guided by the probability of Poisson distribution, which is calculated using the number of mutations involved in certain pathways in PAR and IA samples. The Poisson distribution is performed in R 3.6.3 using ppois() function. The KEGG pathway information of human proteome was downloaded from the KEGG website (www.genome.jp) and the human GO biological pathway and molecular function information were retrieved from the Uniprot website (www.uniprot.org).

### EGFR Protein Structural Analysis

EGFR protein structure was retrieved from the Protein Data Bank (www.rcsb.org) using entry 1M17. The protein structures and mutated residues were illustrated by Pymol software (The PyMOL Molecular Graphics System, Version 1.2r3pre, Schrödinger, LLC.) using “cartoon” and “surface” functions.

### Comparison With TCGA Mutation Data

The genomic mutation information in 32 types of cancers was retrieved from the TCGA (The Cancer Genome Atlas) (13) *via* Xena platform from University of California Santa Cruz. The number of mutations in samples were compared with the TCGA genomic mutation data in each types of cancer, and a paired Wilcoxon test was performed to evaluate the difference between each types of samples.

### Protein-Protein Interaction Network (PPIN) Analysis

The protein-protein interaction information of mutated genes was retrieved from BioGrid (The Biological General Repository for Interaction Datasets) database (14), and the PPIN in each types of samples were illustrated using Cytoscape (15).

### Statistics

All the statistics are performed by RStudio (www.rstudio.com) using R version 3.6.1, and a p-value cutoff at 0.05 is used in all the analysis.

## Results

### Characterization of Pulmonary Nodule Samples

**Figure 1:**
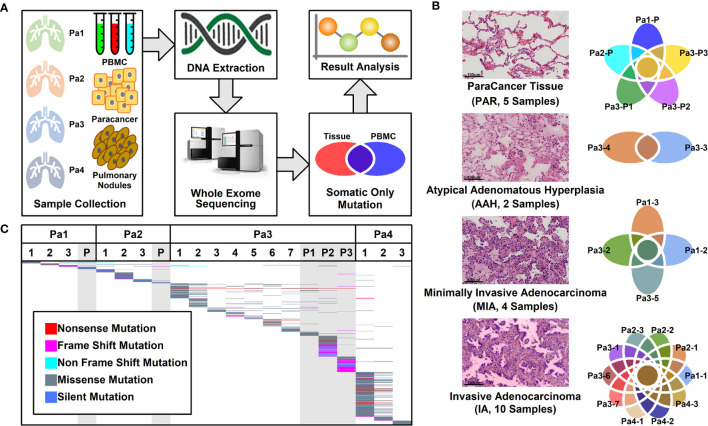
Pulmonary nodule sample selection and whole exome sequencing. **(A)**. Schematic illustration of research design. Twenty-one tissue samples and PBMC samples were collected. After DNA extraction and whole exome sequencing (WES), somatic mutations in different tissues were further analyzed; **(B)** Histological characteristic representation of samples and patient IDs with histopathological stages; **(C)** Summary of WES results across all samples. Each different-colored line represents a different type of mutations, and the scattered lines represent the shared mutations between different samples.

A total of 16 nodules with different stages of neoplasia and five para-cancer (PAR) tissues from four patients diagnosed as multiple pulmonary nodules (MPNs) were collected during surgery. After DNA extraction, whole exome sequencing (WES) was performed on these samples and peripheral blood mononucleated cells (PBMCs) of patients. The somatic mutations were retrieved after the filtration against germ-line mutations through a comparison with PBMC mutations, as illustrated in **Figure 1A**.

Among these 21 samples, two were in atypical adenomatous hyperplasia (AAH) stage, four in minimally invasive adenocarcinoma (MIA) stage, 10 in invasive adenocarcinoma (IA) stage, and five were PAR tissues. The histological characteristics of samples together with their IDs from each stage were presented in **Figure 1B**. The locations of all the lesions were indicated in the CT images and the histological image of each tissue were shown in **Supplemental Figure 1**.

### Mutational Analysis of Whole Exome Sequencing Results

**Figure 2:**
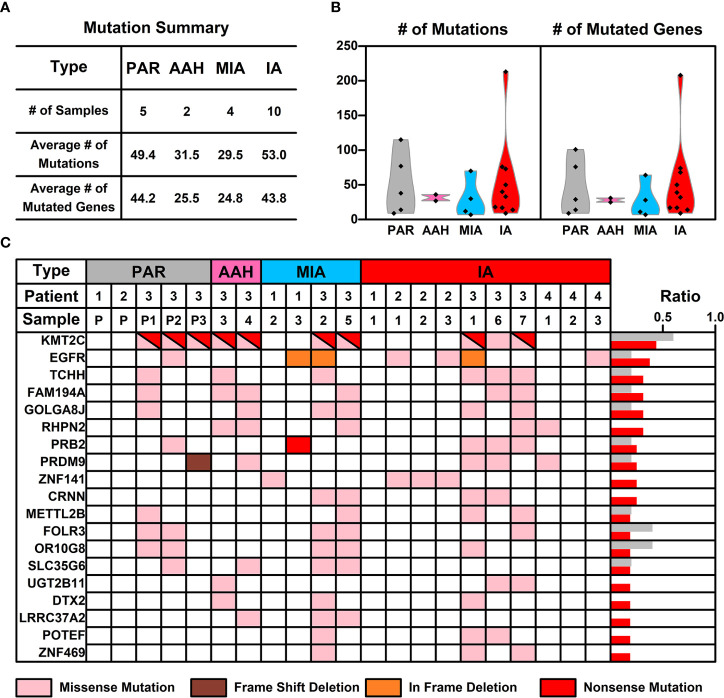
The mutation status of samples from different nodules. **(A)** List of average numbers of mutations/mutated genes in samples from different stages; **(B)** Violin plot representing the mutations (left) and mutated genes (right) in different samples. Each dot represents the number of mutations (non-synonymous mutations, left) and mutated genes (right) in different samples. **(C)** Chart listing hotspot mutated genes occurring in more than two pulmonary nodules. Each colored rectangle represents a mutated gene with different colors indicating different mutation types.

WES was performed on these 21 samples from four patients as well as their PBMCs. The somatic mutations in each sample were summarized in **Figure 1C**. Most of the mutations are sample specific, with a small portion of mutations shared in more than one sample (scattered lines in **Figure 1C**). The number of mutations varies among different samples, with the least number of seven mutations (Pa1-1; Pa-2; Pa2-P) and the highest number of 214 mutations (Pa4-1). The average number of mutations in PAR, AAH, MIA, and IA samples are 49.4, 31.5, 29.5, and 53.0, respectively, and the average number of mutated genes in PAR, AAH, MIA, IA samples are 44.2, 25.5, 24.8, and 43.8, respectively, as listed in **Figures 2A, B**. These results indicated that there is no significant difference in the number of mutations or mutated genes between AAH and MIA samples, but there is a dramatic increase in the number of both mutations and mutated genes in IA samples as compared to the rest of samples.

For mutations on individual gene, missense mutation is the most common one across all types of samples (**Supplemental Figure 2**). As for the mutation types, single nucleotide polymorphism (SNP) is the most common type of mutations across all the samples (**Supplemental Figure 3**), which is consistent with the prevalence of missense mutations; referring to SNV (Single Nucleotide Variant) classes in SNPs, C–>T and G–>A conversions are the top two common SNV types, and these two types of conversions occur most in IA samples (**Supplemental Figure 4**).

### Hotspot Mutated Gene Analysis in Pulmonary Nodules

The frequency of mutated genes in all nodules (AAH, MIA, and IA samples) were further counted and ranked, and the hotspot genes (mutated genes occurred in more than 2 pulmonary nodule samples) were listed in **Figure 2C**. Among these 19 mutated hotspot genes, *KMT2C* (with both missense and nonsense mutations) gene mutation has the highest overall frequency, however, this mutation was only found in the Pa3 samples (including PAR samples); *EGFR* gene mutation, with two types of mutations (missense and in frame deletion mutations), occurs in all four patients and two types of pulmonary samples (MIA and IA); *PRB2* gene mutation, with two types of mutations (missense and nonsense mutations), occurs in two of the patients and two types of pulmonary samples (MIA and IA). Interestingly, some of these hotspot genes with mutation type changes in different types of samples were found and will be further discussed in the following sections.

An interesting phenomenon, as discovered in our study, as well as in many other studies on pulmonary nodules, is the low concurrence of mutated genes between different samples. For example, mutated EGFR, the gene with the highest frequency (KMT2C is treated as specific in patient 3, and hence not counted here), only occurs in 37.5% of pulmonary nodules (6 out of 16 samples). The low concurrence of mutated genes indicates the high genetic diversity of pulmonary nodules, and hence we took a step back and tried to explore the commonality among pulmonary nodule samples from a perspective of signaling pathways and cell functions.

### Converging Tendency Towards Functions or Pathways Related to Malignant Transformation in Pulmonary Nodules

**Figure 3:**
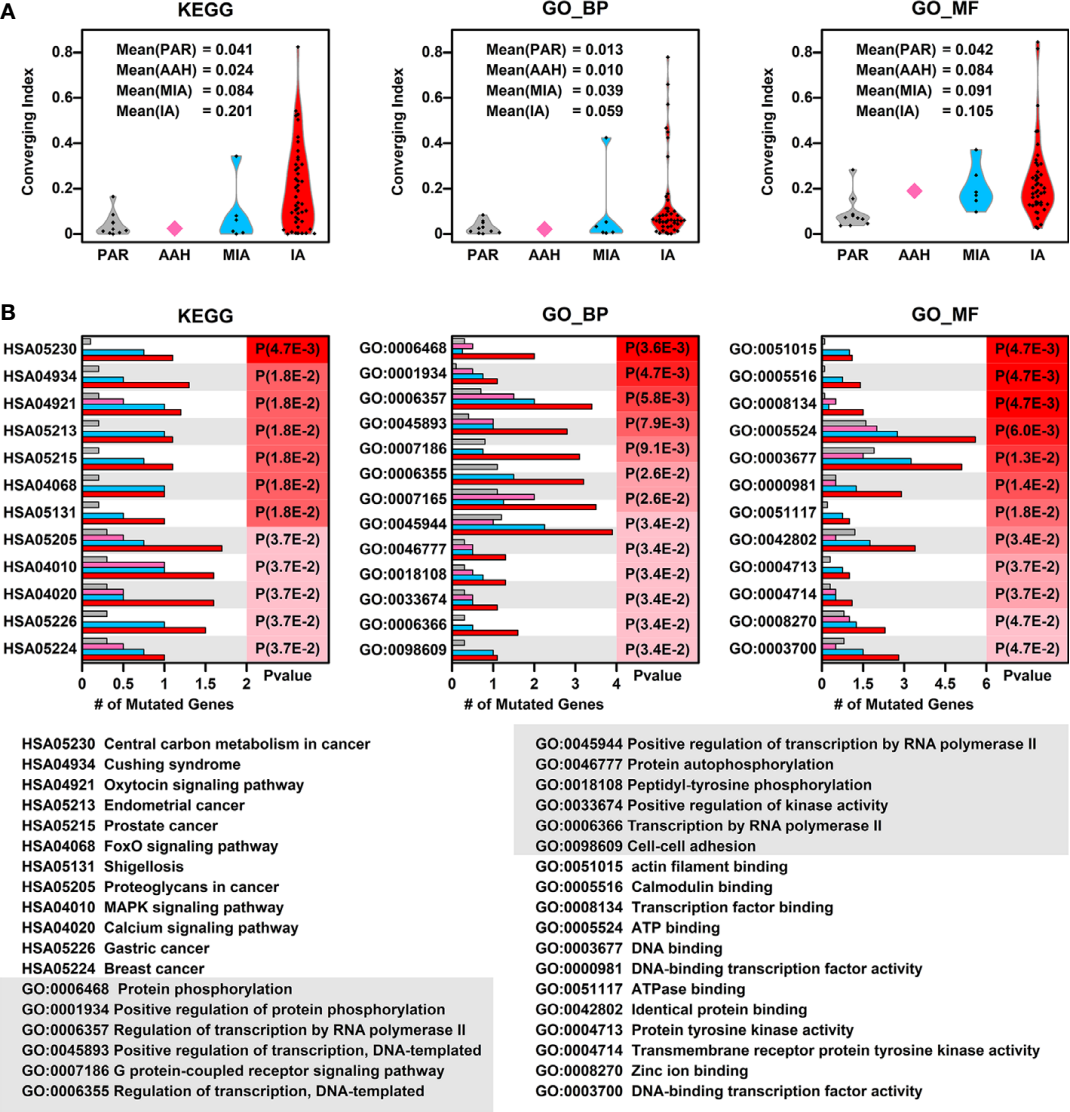
Converging status of samples from different nodules. **(A)** The converging ratio of KEGG pathways (left), GO_BP (GO biological pathways, middle), and GO_MF (GO molecular functions, right) in samples from different nodules. The converging ratio is defined in Materials and Methods with higher converging index representing the higher tendency of mutated genes converging into the same pathway or function; **(B)** The converged KEGG pathways (left), GO_BP (middle), and GO_MF (right) with the number of mutated genes in samples from different stages (PAR, grey; AAH, pink; MIA, cyan; IA, red) and their names (bottom). The converged items were ranked based on the probability calculated by Poisson distribution using the number of mutated genes in PAR samples and IA samples.

To explore whether there is a converging tendency of mutated genes in samples at different nodules, we introduced a converging index, which is defined as the overlapping of signaling pathways or cell functions, including KEGG pathways, GO Biological Pathways (GO_BP), and GO Molecular Functions (GO_MF), in which the mutated genes are involved between two samples (detailed in Materials and Methods). The higher converging index between two samples, the higher probability of two samples with same pathways or cell function got affected (pathway or cell function related genes got mutated). To compare the converging status between samples at different stages, we calculated the converging index for KEGG, GO_BP, and GO_MF between samples within each nodules and the results are shown in **Figure 3A** (there are only two samples in AAH stage hence only one converging index was generated).

In all three types of pathway or cell function terms (KEGG, GO_BP, and GO_MF), the average converging indexes of lesions are higher than those of PAR tissues, except for the indexes in AAH samples, which might be caused by the limitation of sample numbers. Moreover, the indexes increase with the developmental stages of pulmonary nodules (AAH<MIA<IA).

To further explore the converged status of nodular samples at different stages, we retrieved all converged functions/pathways, ranked them based on the enrichment levels (detailed in Materials and Methods) (**Figure 3B**). Among the top 12 converged KEGG pathways, most of them are cancer related or important signaling pathways such as FoxO/MAPK/Calcium pathways; among the top 12 converged GO biological pathways, most of them are involved in transcription regulation and protein phosphorylation, the aberrant functions of these pathways have been shown directly related to lung cancer development in many studies (16). Moreover, GO:0098609 Cell-cell adhesion, which is directly related to epithelial–mesenchymal transition (EMT), is also converged in lesions, indicating that the cell-cell adhesion ability has been affected in the pathogenesis of LUAD and might contribute to metastasis. Among the top 12 converged GO molecular functions, many of them are related to regulation of transcription, which is in consistent with GO_BP results. In addition, GO:0004713, Protein tyrosine kinase activity and GO:0004714 Transmembrane receptor protein tyrosine kinase activity, that are directly involved in cancer-related signaling, have also been affected in pulmonary neoplasia. These findings implicate that multiple cell function disorders in these early stage lesions might trigger and/or accelerate the development of LUAD.

### Effects of Mutation on the Function of the Proteins Encoded by Hotspot Genes

**Figure 4:**
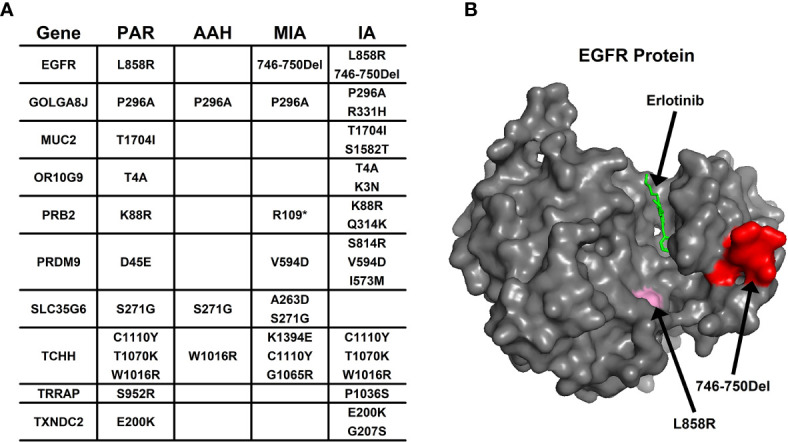
The mutation change in the same gene from different pulmonary nodules. **(A)** List of genes with different mutation in para-cancer tissues (PAR) and pulmonary nodule tissues (AAH, MIA and IA); **(B)** The 3D surface structure of EGFR in complex with Erlotinib. The different types of mutations are highlighted in pink and red colors.

Aside from converging tendency of mutated genes in cancer-related cellular functions and pathways, we also investigated the effect of mutation on the function of hotspot genes at the protein level. The changes of mutation types in pulmonary nodules were compared with mutations in PAR tissues listed in **Figure 4A**. For example, in EGFR protein, there is a point mutation (L858R) in PAR tissues and IA samples; another mutation type, a deletion of 5 amino acids (746-750Del), occurs in both MIA and IA samples. 746-750Del locates in Erlotinib binding region (highlighted in red, **Figure 4B**), and the deletion of this beta3-alphaC loop region has been previously reported in Erlotinib-resistant patient (17).

In PRB2 protein, there is a point mutation of K88R in PAR tissues and IA samples; a nonsense mutation (R1098*) occur in MIA and IA samples. The nonsense mutation R1098* is a premature stop codon which might affect the function of PRB2 protein, and might relate to the development of pulmonary nodules.

Missense mutation might also cause dramatic structural changes. For example, in OR10G9 protein, a missense mutation (K3N) occurs in IA samples, leading to conversion of a positively charged amino acid (Lysine) to a polar amino acid (Asparagine). In PRB2 protein, a missense mutation (Q314K) occurs in IA samples, leading to conversion of a polar amino acid (Glutamine) to a positively charged amino acid (Lysine). In PRDM9 protein, two missense mutations occur in MIA and IA samples (V594D; S814R) leading to conversion of a non-polar amino acid (Valine) to a negatively charged amino acid (Aspartic acid) and a polar amino acid (Serine) to a positively charged amino acid (Arginine). In SLC35G6 protein, a missense mutation occurs in MIA samples (A263D), leading to conversion of a non-polar amino acid (Alanine) to a negatively charged amino acid (Aspartic Acid). In TCHH protein, a missense mutation occurs in MIA samples, (K1394E) leading to conversion of a positively charged amino acid to a negatively charged amino acid (Glutamic Acid). In TXNDC2 protein, a new missense mutation (G207S) occurs in IA samples, leading to conversion of a nonpolar amino acid (Glycine) to a polar amino acid (Serine).

### Pulmonary Nodule Mutated Genes

**Figure 5:**
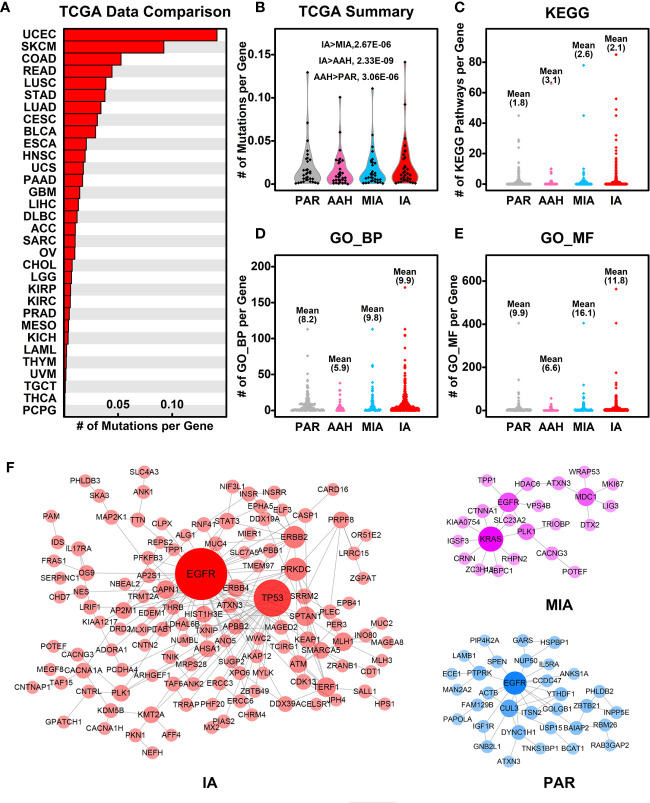
Association of mutated genes in pulmonary nodules and other cancer genes. **(A)** The number of mutations per gene in different types of cancers. The mutated genes in IA samples were used here, and the mutation data from different types of cancers were retrieved from TCGA database; **(B)** The summary of mutations per gene in different pulmonary nodules compared with different types cancers. With the development of pulmonary nodules, the chances of pulmonary nodule related genes that are mutated in different types of cancers increased; **(C–E)** The number of KEGG pathways **(C)**, GO_BP **(D)**, and GO_MF **(E)** in which each mutated gene involved is plotted in different nodules. With the development of pulmonary nodules, the average number of mutated gene involved pathways/functions increases, indicating the functional importance of mutated genes; **(F)** The PPI networks generated using mutated genes from PAR, MIA, and IA samples.

To explore the association of cancer related genes and mutated genes in different pulmonary nodules screened in WES, we retrieved the mutational data of these genes in 32 types of cancers from TCGA database, and compared the average number of mutations per gene among different types of cancers. Among 32 types of cancers, IA-related mutated genes have the highest number of mutations per gene in UCEC (Uterine corpus endometrial carcinoma). LUSC (Lung squamous cell carcinoma) and LUAD (Lung adenocarcinoma) ranks among the top #5 and #7, respectively of these 32 types of cancers, indicating the IA-related mutated genes have a higher mutation rate in lung cancers comparing to other types of cancers (**Figure 5A**). Among the four stages of nodules, pulmonary nodule related genes have a higher mutation rate in all types of cancers in comparison with PAR-related genes, and the mutation rate increases with the development of pulmonary nodules (IA>MIA, IA>AAH, AAH>PAR) (**Figure 5B**).

To explore the functional importance of pulmonary nodule related mutated genes, we calculated the number of each mutated gene involved in KEGG pathways (**Figure 5C**), GO_BP (**Figure 5D**), and GO_MF (**Figure 5E**). The rationale behind this is that the higher the number of genes involved in the pathways/functions, the more important role one gene could play, and hence the mutation could make bigger impact on cell activities. In general, the average numbers of mutated genes involved in pathways/functions are higher in pulmonary nodules than that of PAR-related mutated genes, suggesting the increased functional importance of mutated genes in pulmonary nodules.

We further constructed the protein-protein interaction (PPI) networks generated by mutated genes from different pulmonary nodules (**Figure 5F**) and compared them with each other (the PPI network of AAH-related genes was not shown due to limited number of mutated genes). The PPI network generated by IA-related mutated genes is more complex when compared with MIA or PAR nodules, indicating a complex function affected in IA samples. The complexity of PPI network could contribute to the explanation of development of pulmonary nodules.

## Discussion

MPNs is defined as more than one solid nodules in the lung of a single patient. Pathologically, MPNs are commonly identified as AAH, AIS, MIA, and IA. They represent an ideal model of deciphering the clonal evolution of early stage lung adenocarcinoma. Understanding the gene mutations in MPNs holds great promise for prevention and treatment strategy of lung adenocarcinoma.

Accumulating mutations as well as genes were identified by using next generation sequencing on MPNs samples (6, 9, 18). However, these studies mainly used single gene change-directed data analytic strategy and the clinical significance of these findings are largely limited (19). To overcome the limitations of current genome-based data, we analyzed the somatic mutations identified in the WES data from 21 MPNs followed by a cell function and pathway converging-directed analysis. Based on this function-directed analysis, we found that the extent of protein structural changes caused by different mutations as well as the amounts of affected pathways/functions in all lesions are positively correlated with the degree of malignancy, indicating that during the malignant evolution, comprehensive mutations in several key signaling pathways other than single gene are accumulated, which might endow certain cell populations more aggressive phenotype in terms of accelerated proliferation rates and the malignant progression of lesions.

Among the mutated genes identified in all samples, KMT2C other than EGFR was found in 10 out of 21 lesions. This result is in consistent with the recently reported finding from cell line experiments, indicating that KTM2C might be a novel driver gene correlated with KRAS mutation during the development of early stage lung adenocarcinoma (20). However, KMT2C mutation is only found in one of four patient and only one IA out of all 10 samples harbors a missense mutation, all other samples in particular adjacent normal samples harbored with nonsense mutation. Moreover, many of these mutations including those in EGFR were also identified in adjacent normal tissues when germ-line mutations were filtered during the process of analysis. These findings indicate the complicated background of cancer lesions and the clinical significance of single gene mutation in samples except of cancerous tissues should be carefully reevaluated. On the contrary, EGFR mutation are mainly found in malignant lesions, among which in frame deletion is the major mutational pattern. Similar results were obtained in the amounts and patterns of many other gene mutations in this study.

The KEGG signaling pathway analysis indicates that varied mutated genes tend to converge into signaling pathways related to pivotal cell growth regulation in malignancy in comparison with adjacent normal samples. This converging trend seems to be more obvious in IA than in MIA, indicating a cancer-like microenvironment forced selection of functional mutations during the origination and development of early stage lung adenocarcinoma. Considering the similar number of mutations/mutated genes in PAR and IA samples, the increased converging index indicating mutated genes tend to focus on certain functions/pathways, or only cells with certain impaired functions can survive under these microenvironments. Among the top 12 converged KEGG pathways, most of them are cancer related or important signaling pathways such as FoxO/MAPK/Calcium pathways. Among the top 12 converged GO biological pathways, most of them are involved in transcription regulation and protein phosphorylation, the aberrant functions of these pathways have been shown directly related to cancer development in many studies. Moreover, the levels of protein structural changes caused by mutations as well as the extent of mutations in these converged pathways are higher in IA as compared with that in MIA tissues, both of which are higher in malignant lesions than in adjacent normal tissues, indicating that these pathways may be responsible for the malignant evolution of pulmonary nodules.

Although whole transcriptional sequencing data from other patients might be available, lacking in-patient paired validation in protein expression levels is the major limitation of exploring WES data in MPNs studies. To circumvent this problem, we used a protein structure predicting strategy to evaluate the functional significance of hot spot gene mutations that are related to carcinogenesis and target therapy resistance (as listed in **Figure 4A**) (21). As shown in our results, 746-750 Del mutation of EGFR was identified, which might induce erlotinib resistance in NSCLC patients (17). PRB2, a tumor suppressor (22, 23), a premature stop codon was identified in our study, suggesting that its down-regulation and dysfunction might occur in patients. The mutation of TCHH is found to be related to platinum resistance in gastric carcinoma (24). In our predicting results, the conversion from positively charged amino acid to negatively charged amino acid (K1394E) of TCHH induced by missense mutation might affect its protein stability as well as the prognosis of this patient (25). Down regulation of TXNDC2, an antioxidant enzyme is directly related to cisplatin resistance in many cancer cells (26, 27). In our findings, conversion from nonpolar amino acid to polar one (G207S) in protein structure is also predicted. These findings are coincide with previous evidences on the mutation analysis in a serial of studies (28–31). Besides, in comparison with The Cancer Genome Atlas database (TCGA), most of mutated genes in MIA and IA identified in this study are genes functionally related to the development of lung cancers.

In this study, the findings by function-directed analysis of WES data from MPNs suggested that a group of functionally converged mutations rather than a single driver gene mutation might be the trigger of the origination and malignant progression of lung adenocarcinoma. Our results indicated that other than well-known genes such as EGFR, KRAS, and TP53, mutations in other genes involved in relating pathways (as listed in **Figure 3B**) deserve more attention, and an involvement of these genes might be necessary in the future screening of early stage lung adenocarcinoma. Besides, our results shed some new insights as well as new strategy to decipher the early development of lung cancer, and also provide many candidate genes which might be used as potential drug targets in the future treatment of lung adenocarcinoma.

## Data Availability Statement

The data presented in the study are deposited in Genome Sequence Archive for Human (“https://bigd.big.ac.cn/gsa-human/”) repository, accession number HRA000465.

## Ethics Statement

The present study was approved by the Ethics Committee of Shenzhen People’s Hospital. The patients/participants provided their written informed consent to participate in this study. Written informed consent was obtained from the individual(s) for the publication of any potentially identifiable images or data included in this article.

## Author Contributions

CZ, S-WD, CQ, and G-sW conceived the research idea. CZ, S-WD, ZC, and CQ prepared and wrote the manuscript. D-CL, JXu, SL, PZ, and JAW collected the clinical samples. RL, JXi, YY, JGW, LD, and XW analyzed the WES data. SD, YF, YC, and CZ revised the manuscript. All authors contributed to the article and approved the submitted version.

## Funding

This study is funded by The Science and Technology Foundation of Shenzhen (JCYJ20180305164128430); the International Cooperation Foundation of Shenzhen (GJHZ20180928171602104); the Shenzhen Economic and Information Committee “Innovation Chain and Industry Chain” integration special support plan project (20180225112449943); the Shenzhen Public Service Platform on Tumor Precision Medicine and Molecular Diagnosis; Research Grants Council of the Hong Kong Special Administrative Region, China (Project No. CityU 21101115, 11102317, 11103718, 11103619, R4017-18, C4041-17GF), a grant from Guangdong Basic and Applied Basic Research Foundation (Project No. 2019B030302012), Young Scientists Fund of the National Natural Science Foundation of China (81802384); International innovation team for early diagnosis and precise treatment of lung cancer (2016, KQTD2016113015442590); Scientific Research Project of Taikang Xianlin Drum Tower Hospital (TKKY20193805).

## Conflict of Interest

The authors declare that the research was conducted in the absence of any commercial or financial relationships that could be construed as a potential conflict of interest.
