# Supplementary material for: Mutational Pattern in Multiple Pulmonary Nodules Are Associated With Early Stage Lung Adenocarcinoma
Source: Front Oncol. 2021 Feb 19;10:571521. doi: 10.3389/fonc.2020.571521 (PMC7934775; doi:10.3389/fonc.2020.571521)
Supplement: Supplementary file 1 [file DataSheet_1.docx]

**
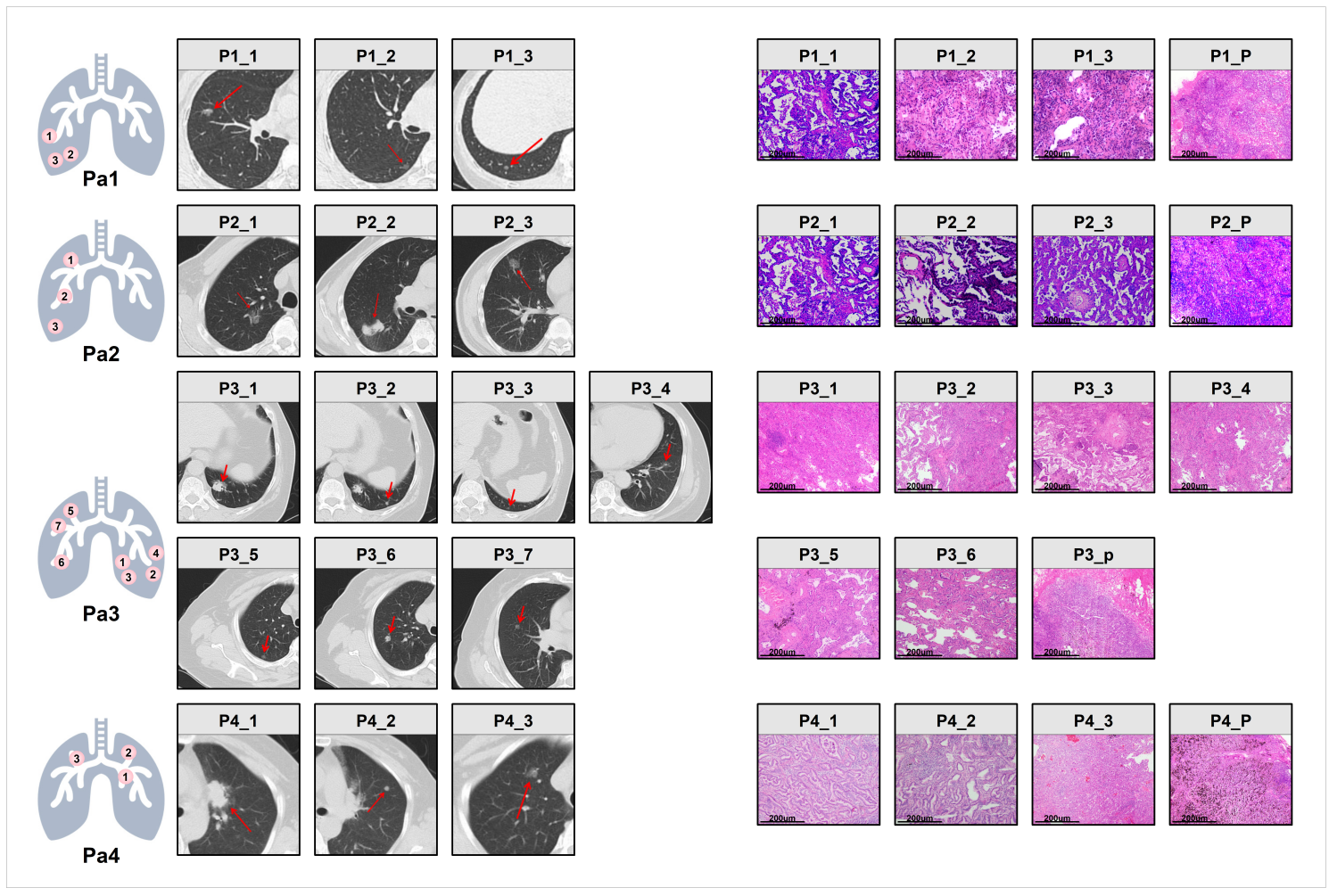
**

**Supplementary Figure 1.** **The CT and histological image of selected samples**


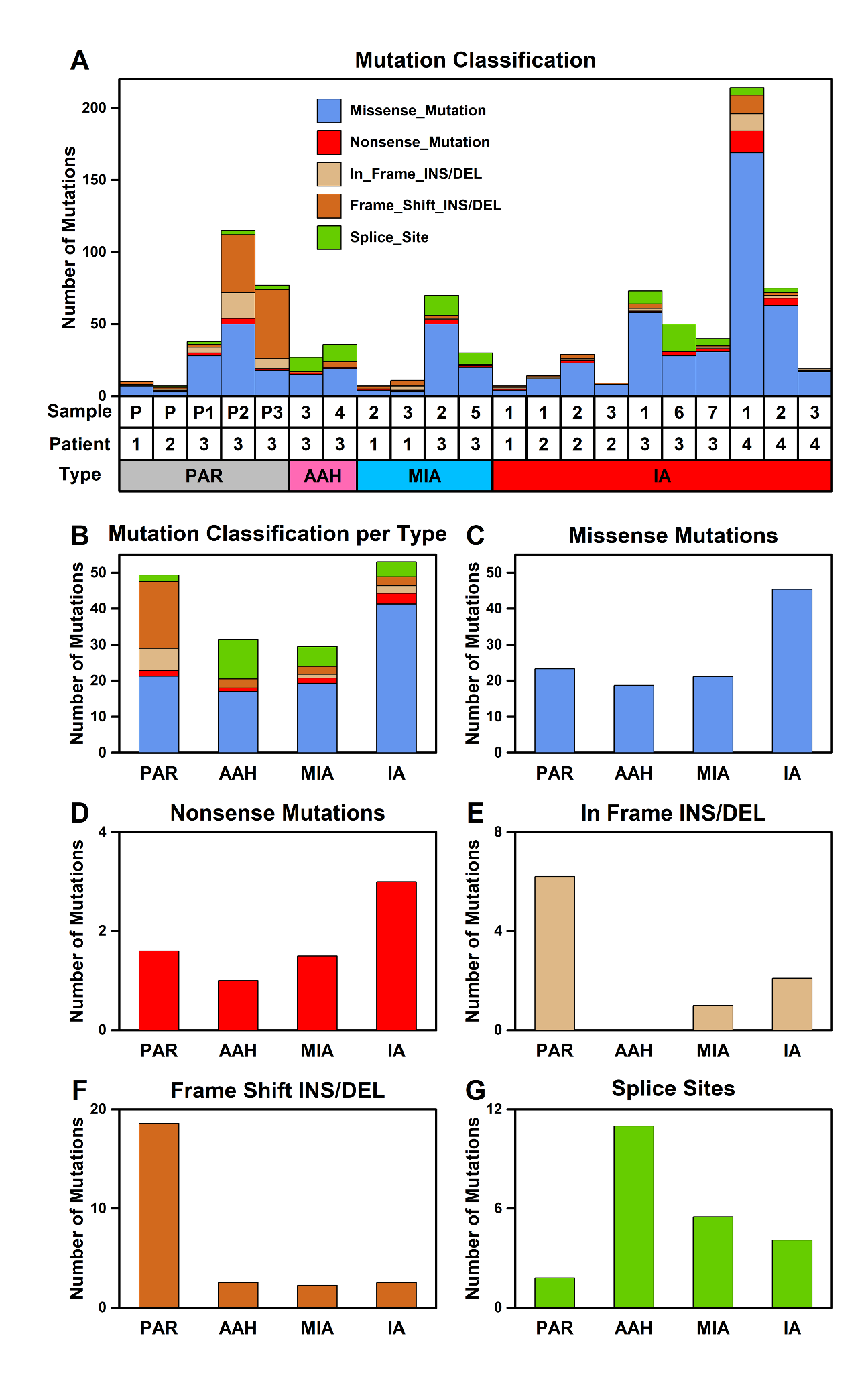


**Supplementary Figure 2. Mutation classification of all samples.** (A) Bar plot showing the number of different classes of mutations in 21 samples; (B) Bar plot showing the number of different classes of mutations in four types of samples; (C-G) Bar plot showing the average number of different classes of mutations in four types of samples.


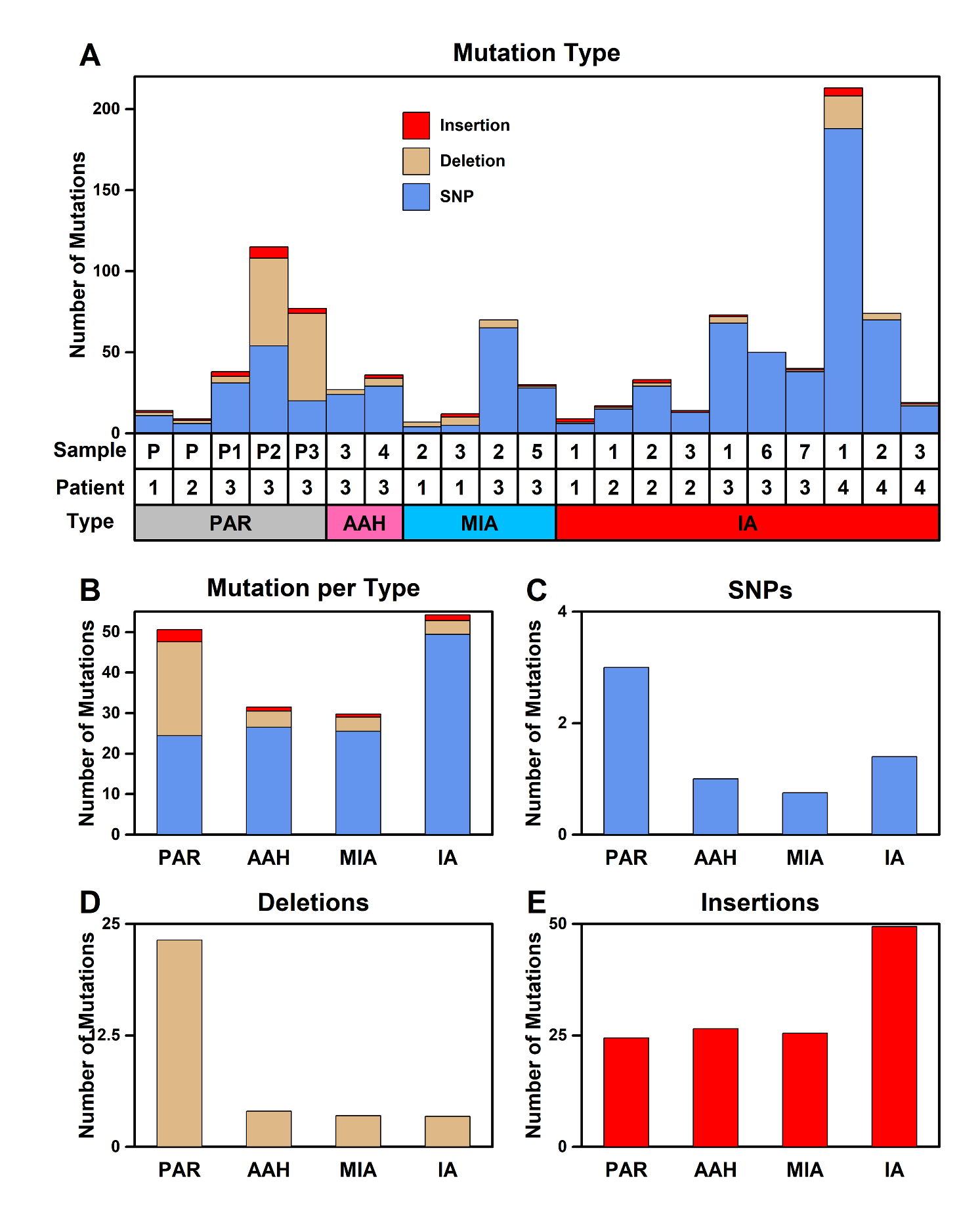


**Supplementary Figure 3.** **Mutation types of all samples.** (A) Bar plot showing the number of different types of mutations in 21 samples; (B) Bar plot showing the number of different types of mutations in four types of samples; (C-E) Bar plot showing the average number of different types of mutations in four types of samples.


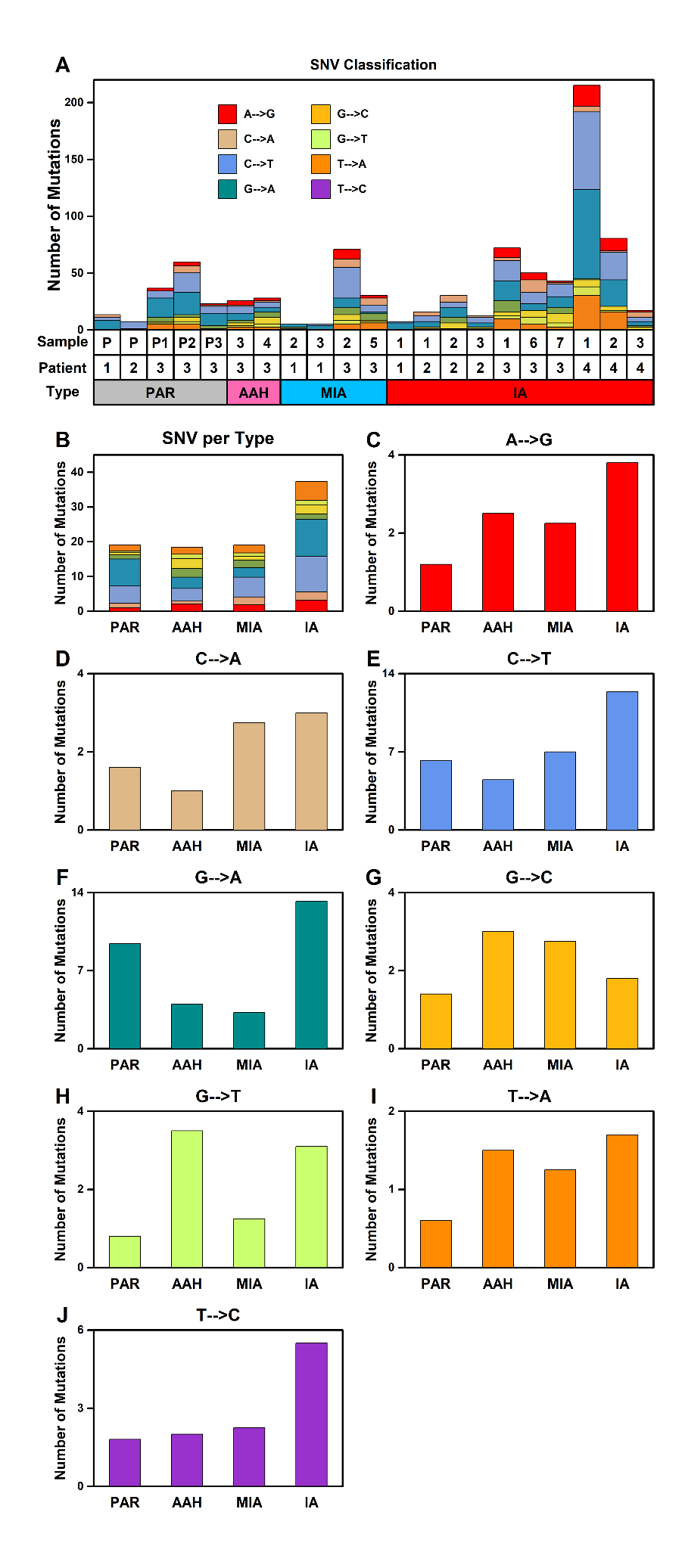


**Supplementary Figure 4.** **SNV types of all samples.** (A) Bar plot showing the number of different types of SNVs in 21 samples; (B) Bar plot showing the number of different types of SNVs in four types of samples; (C-J) Bar plot showing the average number of different types of SNVs in four types of samples.
